# PEER-LED EXERCISE RESULTS IN IMPROVED SLEEP EFFICIENCY WITHOUT CONCURRENT CHANGES IN MOOD

**DOI:** 10.1093/geroni/igae098.3347

**Published:** 2024-12-31

**Authors:** Chitra Banarjee, Kworweinski Lafontant, Jethro Raphael Suarez, Joon-Hyuk Park, David Fukuda, Jeffrey Stout, Nichole Lighthall, Ladda Thiamwong

**Affiliations:** University of Central Florida, Orlando, Florida, United States; University of Central Florida, Orlando, Florida, United States; University of Central Florida; University of Central Florida, Orlando, Florida, United States; University of Central Florida, Orlando, Florida, United States; University of Central Florida, Orlando, Florida, United States; University of Central Florida, Orlando, Florida, United States; University of Central Florida, Orlando, Florida, United States

## Abstract

Sleep is an important physiological process for recovery and regulation of homeostatic processes in the human body. Changes in sleep have additionally been shown to affect mood, including anxiety and depression. The present study implements multimodal assessment of sleep and mood using the wearable FitBit® Charge 5, as well as subjective surveys, the PHQ-9 and GAI, evaluating anxiety and depression, respectively. The study sample included 108 community-dwelling older adults (mean age = 74.19 years ± 6.96; 89.8% female). Of these adults, 58 participated in weekly peer-led exercise and exercise, (strength-training and walking) at least 30 mins twice a week for eight weeks. Participants wore a FitBit® for a mean of 76.5 days ± 59.24, including at night. Using the FitBit®, the study evaluated time asleep and sleep efficiency, calculated based on the time asleep, deep and REM sleep, and restoration. Participants undergoing intervention exhibited a significant increase in both sleep duration (p< 0.001; intervention=297.2 minutes, control=274.8 minutes) and efficiency (p< 0.001; intervention=93.6, control=92.2), as evaluated by Wilcoxon tests. However, subjective assessments of anxiety (p=0.288) and depression (p=0.986) did not reveal significant differences. These results indicate that peer-led exercise results in a significant improvement in sleep, which is consistent with previous research. The lack of concordant results in mood suggests that more intensive exercise may be necessary to improve mood. Additionally, the study was limited by individual variability in FitBit® wear time. Future research should evaluate the long-term effects of increasing the time and intensity of peer-led exercise on sleep and mood.

